# The respiratory syncytial virus prefusion F protein vaccine attenuates the severity of respiratory syncytial virus‐associated disease in breakthrough infections in adults ≥60 years of age

**DOI:** 10.1111/irv.13236

**Published:** 2024-02-03

**Authors:** Desmond Curran, Sean Matthews, Eliazar Sabater Cabrera, Silvia Narejos Pérez, Lina Pérez Breva, Mika Rämet, Laura Helman, Dae Won Park, Tino F. Schwarz, Isabel Maria Galan Melendez, Axel Schaefer, Nathalie Roy, Brigitte Stephan, Daniel Molnar, Lusine Kostanyan, John H. Powers, Veronica Hulstrøm, Members of the AReSVi‐006 Study Group, Mark Adams, Michael Adams, Elaine Jacqueline Akite, Ingrid Alt, Charles Andrews, Rafaelle Antonelli‐Incalzi, Asmik Asatryan, Eugene Athan, Ghazaleh Bahrami, Elena Bargagli, Qasim Bhorat, Paul Bird, Przemyslaw Borowy, Celine Boutry, Carles Brotons Cuixart, David Browder, Judith Brown, Erik Buntinx, Donald Cameron, Laura Campora, Kenneth Chinsky, Melissa Choi, Eun‐Ju Choo, Delphine Collete, Maria Corral Carrillo, Marie‐Pierre David, Matthew G. Davis, Magali de Heusch, Ferdinandus de Looze, Marc De Meulemeester, Ferdinando De Negri, Nathalie De Schrevel, David DeAtkine, Viktoriya Dedkova, Dominique Descamps, Nancy Dezutter, Peter Dzongowski, Tamara Eckermann, Brandon Essink, Karen Faulkner, Robert Feldman, Murdo Ferguson, Laurence Fissette, Gregory Fuller, Ivan Gentile, Wayne Ghesquiere, Doria Grimard, Olivier Gruselle, Scott Halperin, Amardeep Heer, Andre Hotermans, Michael G. Ison, Tomas Jelinek, Jackie Kamerbeek, Hyo Youl Kim, Murray Kimmel, Mark Koch, Satu Kokko, Susanna Koski, Shady Kotb, Antonio Lalueza, Joanne M. Langley, Dong‐Gun Lee, Jin‐Soo Lee, Isabel Leroux‐Roels, Muriel Lins, Johannes Lombaard, Akbar Mahomed, Mario Malerba, Celine Marechal, Federico Martinon‐Torres, Jean‐Benoit Martinot, Cristina Masuet‐Aumatell, Damien McNally, Carlos Eduardo Medina Pech, Jorge Mendez Galvan, Narcisa Elena Mesaros, Dieter Mesotten, Essack Mitha, Kathryn Mngadi, Beate Moeckesch, Barnaby Montgomery, Linda Murray, Rhiannon Nally, Joseph Newberg, Paul Nugent, Dolores Ochoa Mazarro, Harunori Oda, Aurelie Olivier, Maurizio Orso, Jacinto Ortiz Molina, Tatiana Pak, Alberto Papi, Meenakshi Patel, Minesh Patel, Anna Maria Pedro Pijoan, Merce Perez Vera, Alberto Borobia Perez, Claudia Pileggi, Fabrizio Pregliasco, Carol Pretswell, Dean Quinn, Michele Reynolds, Viktor Romanenko, Jeffrey Rosen, Belen Ruiz Antoran, Hideaki Sakata, Joachim Sauter, Izabela Sein Anand, Jose Antonio Serra Rexach, David Shu, Andres Siig, William Simon, Svetlana Smakotina, Katie Steenackers, Silvio Tafuri, Kenji Takazawa, Guy Tellier, Wim Terryn, Leslie Tharenos, Nick Thomas, Nicole Toursarkissian, Benita Ukkonen, Noah Vale, Marie Van der Wielen, Pieter‐Jan Van Landegem, Richard N. van Zyl‐Smit, Carline Vanden Abeele, Celine Verheust, Lode Vermeersch, Miguel Vicco, Francesco Vitale, Olga Voloshyna, Judith White, Seong‐Heon Wie, Jonathan Wilson, Pedro Ylisastigui

**Affiliations:** ^1^ GSK Wavre Belgium; ^2^ Freelance c/o GSK Wavre Belgium; ^3^ CAP Centelles Barcelona Spain; ^4^ Vaccine Research FISABIO‐Public Health Valencia Spain; ^5^ Finnish Vaccine Research Tampere Finland; ^6^ Department of Clinical Medicine George Washington University School of Medicine & Health Sciences Washington District of Columbia USA; ^7^ Korea University Ansan Hospital Ansan Republic of Korea; ^8^ Institute of Laboratory Medicine and Vaccination Centre Klinikum Würzburg Mitte Würzburg Germany; ^9^ Hospital Universitario Fundacion Alcorcon Madrid Spain; ^10^ Medizentrum Essen Borbeck Essen Germany; ^11^ Medicor Research Greater Sudbury Canada; ^12^ SGS proDERM Schenefeld Germany

**Keywords:** acute respiratory infections, older adults, patient‐reported outcome, quality of life, respiratory syncytial virus

## Abstract

**Background:**

Respiratory syncytial virus (RSV) is a contagious pathogen causing acute respiratory infections (ARIs). Symptoms range from mild upper respiratory tract infections to potentially life‐threatening lower respiratory tract disease (LRTD). In adults ≥60 years old, vaccine efficacy of a candidate vaccine for older adults (RSVPreF3 OA) was 71.7% against RSV‐ARI and 82.6% against RSV‐LRTD (AReSVi‐006/NCT04886596). We present the patient‐reported outcomes (PROs) from the same trial at the end of the first RSV season in the northern hemisphere (April 2022).

**Methods:**

In this phase 3 trial, adults aged ≥60 years were randomized (1:1) to receive one dose of RSVPreF3 OA vaccine or placebo. PROs were assessed using InFLUenza Patient‐Reported Outcome (FLU‐PRO), Short Form‐12 (SF‐12), and EuroQol‐5 Dimension (EQ‐5D) questionnaires. Peak FLU‐PRO Chest/Respiratory scores during the first 7 days from ARI episode onset were compared using a Wilcoxon test. Least squares mean (LSMean) of SF‐12 physical functioning (PF) and EQ‐5D health utility scores were estimated using mixed effects models.

**Results:**

In the RSVPreF3 OA group (*N* = 12,466), 27 first RSV‐ARI episodes were observed versus 95 in the Placebo group (*N* = 12,494). Median peak FLU‐PRO Chest/Respiratory scores were lower in RSVPreF3 OA (1.07) versus Placebo group (1.86); *p* = 0.0258. LSMean group differences for the PF and EQ‐5D health utility score were 7.00 (95% confidence interval [CI]: −9.86, 23.85; *p* = 0.4125) and 0.0786 (95% CI: −0.0340, 0.1913; *p* = 0.1695).

**Conclusions:**

The RSVPreF3 OA vaccine, in addition to preventing infection, attenuated the severity of RSV‐associated symptoms in breakthrough infections, with trends of reduced impact on PF and health utility.

## Graphical abstract

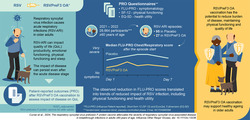

## 1 INTRODUCTION

Respiratory syncytial virus (RSV) is a highly contagious, human pathogen that causes acute respiratory infections (ARIs) and associated symptoms (i.e., acute respiratory disease). Although RSV has long been recognized as a common cause of respiratory infections in children, it has been increasingly acknowledged as an important cause of severe respiratory illness in older adults, leading to substantial morbidity and mortality.^1^, ^2^ In the United States, 1% to 7% of older adults are infected with RSV each year, resulting in an estimated 178,000 RSV‐related hospitalizations and 14,000 RSV‐related deaths among adults aged ≥65 years.^2^, ^3^

RSV circulation is subject to seasonality, with the infection being transmitted either via coughing or sneezing or via direct or indirect contact with nasal or oral secretions from infected people.^4^ Symptomatic RSV infections manifest as generally mild upper respiratory tract disease or as a more severe lower respiratory tract disease (LRTD).^1^ The risk of severe RSV disease is higher in older adults and in adults with chronic cardiopulmonary conditions or with weakened immune system, potentially leading to more serious outcomes, such as pneumonia, exacerbation of an underlying condition (e.g., asthma or chronic obstructive pulmonary disease), and congestive heart failure.^5^

Typically, RSV infections in older adults result in cold‐like symptoms with a median duration of approximately 17 days.^6^ In a recent study that included individuals ≥50 years of age, 52 signs and/or symptoms were reported to be associated with an RSV episode, the most bothersome of which were coughing, trouble breathing, fever or feeling feverish, and body aches or pains.^7^ RSV infection impacted health‐related quality of life (HRQoL), in particular productivity, social activities and relationships, emotional functioning, physical functioning (PF), and sleep. All participants who were working reported major impacts on work, and most individuals reported symptoms lasting beyond the acute disease stage ranging from 1 week to >1 month.^7^ Some older adults with RSV infection, especially hospitalized individuals, demonstrate acute functional decline that may become prolonged and can result in higher level of care at discharge and loss of previous independence.^8^

The GSK RSV candidate vaccine for older adults (RSVPreF3 OA) is a combination of the RSVPreF3 antigen, a recombinant F protein antigen engineered to preferentially maintain its prefusion form, and GSK's Adjuvant System 01_E_ (AS01_E_), an adjuvant system that promotes induction/boosting of antibody and cellular responses to overcome age‐related decline in immunity.^9^ The recombinant RSVPreF3 OA vaccine had 71.7% efficacy (VE) against RSV‐ARI and 82.6% VE against RSV‐LRTD in adults aged ≥60 years in a phase 3 clinical trial (AReSVi‐006/NCT04886596).^9^ Here, we report the patient‐reported outcome (PRO) data from this clinical trial during the first RSV season.

## 2 METHODS

### 2.1 Study design

In this ongoing, phase 3, observer‐blind, multi‐country trial, individuals aged ≥60 years were randomized 1:1 to receive either a single dose of AS01_E_‐adjuvanted RSV prefusion F protein candidate vaccine (RSVPreF3 OA) or placebo at study entry. The primary objective was to demonstrate VE of a single RSVPreF3 OA dose against RSV‐confirmed LRTD during one RSV season. An ARI episode was defined, via a clinical assessment, as the presence of at least two respiratory symptoms/signs (see also tab. S2 in Papi et al.^9^) for at least 24 h, or at least one respiratory and one systemic symptom/sign for at least 24 h, and confirmed by reverse transcriptase polymerase chain reaction (RT‐PCR). Further details on the study design are provided in an article presenting the efficacy and safety results of the trial.^9^

### 2.2 Outcome measures

Patient‐reported assessments included in the trial were performed using the InFLUenza Patient‐Reported Outcome (FLU‐PRO) questionnaire, the Short Form‐12 (SF‐12) health survey, the EuroQol‐5 Dimension (EQ‐5D) questionnaire, the Patient Global Impression of Severity (PGI‐S), and the Patient Global Impression of Change (PGI‐C). These questionnaires focus on symptoms, functioning, health utility, and overall assessment of severity, respectively.

The FLU‐PRO version 2.0 is a 32‐item daily diary assessing influenza signs and symptoms across six body systems (i.e., domains): Nose (four items), Throat (three items), Eyes (three items), Chest/Respiratory (seven items), Gastrointestinal (four items), and Body/Systemic (11 items). Participants were asked to rate each sign or symptom on a 5‐point ordinal scale (see the Supporting Information for more details), with higher scores indicating a greater severity of the sign or symptom. Each domain score was calculated as the mean of all items comprising that domain, with scores ranging from 0 (symptom free) to 4 (very severe symptoms).^10^, ^11^ Similarly, the FLU‐PRO total score was computed as the mean score across all 32 items comprising the instrument. In addition, a score assessing the symptoms associated with the upper respiratory system was computed as the mean score across the 10 items that make up the Nose, Throat, and Eyes domains. Validity of the FLU‐PRO to assess RSV disease in older adults has been demonstrated by Yu et al.^6^ and Curran et al.^7^

The SF‐12 questionnaire is a multi‐purpose health survey with 12 questions covering eight domains (Physical Functioning, Role Physical, Bodily Pain, General Health, Vitality, Social Functioning, Role Emotional, and Mental Health).^12^, ^13^ Scale scores were constructed following the SF‐12 scoring algorithms. For all SF‐12 domains, a higher score indicates a higher level of functioning and/or HRQoL.

The EQ‐5D questionnaire is a health utility instrument including five dimensions: Mobility, Self‐Care, Usual Activities, Pain/Discomfort, and Anxiety/Depression. Participants were asked to grade their extent of problems (no problem, some problems, and severe problems). The combination of answers to the five dimensions results in 243 possible health states, each of which is translated into a health utility score ranging from less than 0 (i.e., a health state worse than death) to 1 (i.e., best possible health state).^14^, ^15^

To estimate the minimum clinically important differences (MCIDs), an anchor‐based approach as recommended by the U.S. Food and Drug Administration, was applied.^16^ The anchor‐based method consists of the estimation of the relationship of the changes in the PRO targeted scores with an external criterion or “anchor” of the outcome, which is being measured, that is, respiratory symptom severity. In the current study, the PGI‐S and PGI‐C were included, as anchor measurements, to estimate MCIDs for the FLU‐PRO domains. The PGI‐S and PGI‐C are two patient‐reported questions on the severity of symptoms and on the change of symptoms, respectively.^17^ The PGI‐S classifies the severity of symptoms into five categories (no symptoms, mild, moderate, severe, and very severe). The PGI‐C classifies the change in symptoms from the previous day into seven categories (much better, somewhat better, a little better, about the same, a little worse, somewhat worse, and much worse).

Duration of an RSV episode was calculated based on clinician‐reported outcomes. The start and end date of each symptom and the presence/absence of each sign were recorded in the electronic case report form. The ARI onset date was defined as the first day when the study participant presented with at least two concomitant ARI symptoms/signs meeting the ARI case definition (see tab. S2 in Papi et al.^9^). The ARI end date was defined as the first day when all ARI symptoms/signs had returned to baseline or diminished significantly as assessed by the investigator. The duration of the episode was calculated as the interval between the start date and the end date. Any medically attended visit during the RSV‐ARI episode was also documented by the investigator. Post hoc analysis of VE against medically attended RSV‐ARI and RSV‐LRTD episodes was conducted.

### 2.3 Timing of assessment

Participants were asked to complete the SF‐12 and EQ‐5D questionnaires at baseline, that is, at the vaccination visit (Figure S1). The FLU‐PRO, PGI‐S, and PGI‐C questionnaires were completed at Visit 2 (i.e., 28–42 days after vaccination). Subsequently, after experiencing at least two ARI symptoms/signs for 24 h, participants had to contact the site staff, to plan an ARI site visit. Participants with suspected ARI were asked to complete the FLU‐PRO, the PGI‐S, and PGI‐C daily from the day of onset until the end of the ARI episode or for a maximum of 14 consecutive days. Participants completed the SF‐12 and EQ‐5D once during the ARI episode while attending the scheduled ARI visit.

### 2.4 Statistical analysis

Efficacy analyses were performed in the modified exposed set (mES) which included all the participants who had received vaccine or placebo and did not report an RSV‐ARI prior to Day 15 after vaccination. All PRO analyses were carried out on all RT‐PCR‐confirmed RSV episodes, further referred to as RSV‐ARI episodes, in the mES cohort. No adjustment for multiplicity was performed.

For each RSV‐ARI episode, the maximum (i.e., peak) score for FLU‐PRO Chest/Respiratory scale and FLU‐PRO upper respiratory scale (i.e., nose, throat, and eyes symptoms), during the first 7 days from the onset of ARI symptoms was calculated. The peak FLU‐PRO scores were compared between study groups using a Wilcoxon non‐parametric test.

Repeated‐measures mixed effects model were fitted to compare the impact of RSV‐ARI on SF‐12 PF, EQ‐5D utility score, and FLU‐PRO total score, between the RSVPreF3 OA and Placebo groups, during the first 7 days from the onset of ARI symptoms. The models were fitted including terms for age category (60–69, 70–79, and ≥80 years of age), region (Asia, Europe, and North America), and study group by timepoint (at baseline and during RSV‐ARI episode) interaction. The least squares mean (LSMean) estimates for timepoint by study group and the difference in LSMeans and associated *p* values were obtained from the model.

To evaluate MCIDs for the FLU‐PRO domains, a post hoc analysis was carried out to estimate the mean daily change in the total FLU‐PRO and FLU‐PRO Chest/Respiratory scores associated with a change in the rating of the PGI‐C and the PGI‐S. A mean daily change in total FLU‐PRO and FLU‐PRO Chest/Respiratory scores corresponding to an improvement in symptom severity of one point in either in PGI‐C or PGI‐S was defined as an MCID.

VE was calculated as 1 minus the relative risk with the use of the conditional exact binomial method based on the Poisson model.^18^

## 3 RESULTS

In total, 26,664 participants were enrolled. Of these, 24,960 were included in the mES. A total of 122 participants had an RSV‐ARI (27 in the RSVPreF3 OA group, *N* = 12,466; 95 in the Placebo group, *N* = 12,494).^9^

**FIGURE 1:**
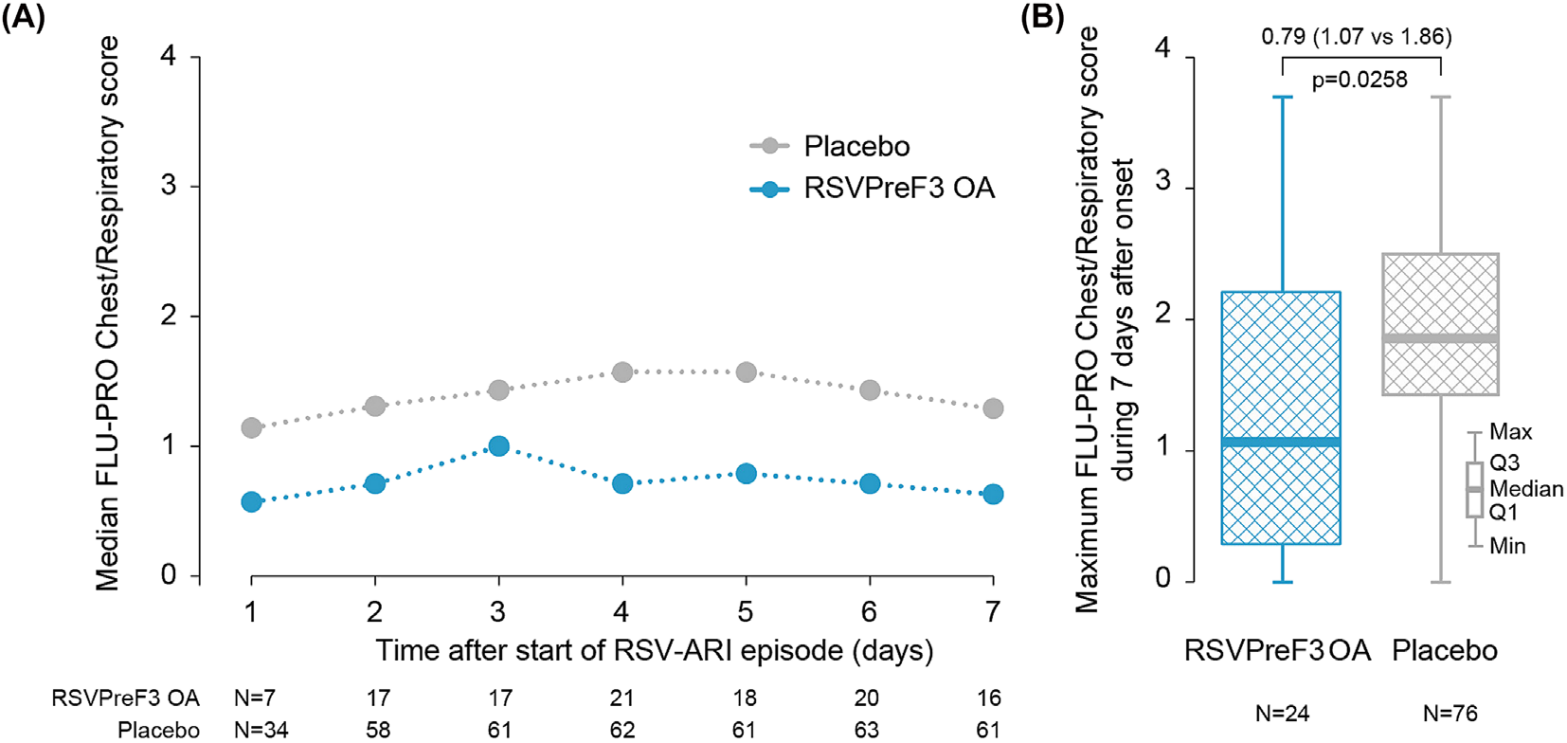
Median daily FLU‐PRO Chest/Respiratory scores and box‐plot distribution of the maximum (peak) FLU‐PRO Chest/Respiratory score by study group (mES RT‐PCR‐confirmed RSV‐ARI cohort). Values shown are the median values of the FLU‐PRO Chest/Respiratory scores on Days 1 through 7 of the RSV‐ARI episode (panel A) and the maximum (peak) FLU‐PRO Chest/Respiratory scores observed over the course of the RSV‐ARI episode (Days 1 through 7) (panel B). A higher score indicates a greater severity of symptoms/problems. *Note*: The minimum and maximum values shown in panel B are across both groups, to maintain blinding of the ongoing study. Data for the other FLU‐PRO domains are presented in Table S3. RSVPreF3 OA, participants receiving RSVPreF3 OA vaccine (27 participants with RSV‐ARI episodes); Placebo, participants receiving placebo (95 participants with RSV‐ARI episodes). FLU‐PRO, InFLUenza Patient‐Reported Outcome; Max, maximum; mES, modified exposed set; Min, minimum; *N*, number of observations; Q1, 25th percentile; Q3, 75th percentile, RSV‐ARI, respiratory syncytial virus–acute respiratory infection; RT‐PCR, reverse transcription polymerase chain reaction.

Overall, 82.0% (100/122) of participants completed at least one FLU‐PRO questionnaire during the first 7 days of the RSV‐ARI episode with similar completion rates in both groups, that is, 24/27 (88.9%) in the RSVPreF3 OA group and 76/95 (80.0%) in the Placebo group (see Figure 1). The completion rates ranged from 33.6% (41/122) on Day 1 to 68.0% (83/122) on Days 4 and 6. The median FLU‐PRO Chest/Respiratory scores were consistently lower in the RSVPreF3 OA group versus the Placebo group for each timepoint between Days 1 and 7 from onset of the RSV‐ARI episode (Figure 1). The median peak FLU‐PRO Chest/Respiratory score observed during the first 7 days was significantly lower in the RSVPreF3 OA group (1.07) versus the Placebo group (1.86), with a *p* value of 0.0258 (Table 1 and Figure 1). The observed difference in median peak FLU‐PRO Chest/Respiratory score between study groups (i.e., 0.79, corresponding to a 42% reduction in the median score) was approximately three times larger than the estimated MCID (0.26; Tables S1 and S2) confirming that the difference between groups represents a decrease in lower respiratory tract symptom severity that was both clinically relevant and statistically significant.

**TABLE 1:** Maximum (peak) FLU‐PRO Chest/Respiratory score during the first 7 days from the onset of the first RT‐PCR‐confirmed RSV‐ARI episode (mES RT‐PCR‐confirmed RSV‐ARI cohort).

Characteristic	RSVPreF3 OA *N* = 27	Placebo *N* = 95	*p* value
Chest/respiratory			
*N* with data	24	76	
Median	1.07	1.86	0.0258
Q1–Q3	0.29–2.21	1.43–2.50	
Mean (SD)	1.32 (1.02)	1.90 (0.93)	
Upper respiratory			
*N* with data	24	76	
Median	1.59	1.40	0.7199
Q1–Q3	0.80–2.20	1.10–1.94	
Mean (SD)	1.56 (0.88)	1.49 (0.73)	

*Note*: A higher score indicates a higher level of symptoms severity.

Abbreviations: FLU‐PRO, InFLUenza Patient‐Reported Outcome; mES, modified exposed set; *N*, number of first RT‐PCR‐confirmed RSV‐ARI episodes; Placebo, participants receiving placebo; Q1, 25th percentile; Q3, 75th percentile; RSV‐ARI, respiratory syncytial virus–acute respiratory infection; RSVPreF3 OA, participants receiving RSVPreF3 OA vaccine; RT‐PCR, reverse transcription polymerase chain reaction; SD, standard deviation.

The median peak FLU‐PRO upper respiratory score observed during the first 7 days was 1.59 in the RSVPreF3 OA group compared with 1.40 in the Placebo group, with a *p* value of 0.7199 (Table 1). Median and mean scores for the peak of the other domains were similar in both study groups (Table S3). In both groups, the Nose and Throat domains had the highest median and mean scores whereas the Gastrointestinal domain had the lowest median and mean scores.

The overall estimated LSMean (standard error [SE]) of the FLU‐PRO total score during the first 7 days of the ARI episode was 0.76 (0.15) in the RSVPreF3 OA group and 0.81 (0.15) in the Placebo group, with an estimated difference of −0.05 (95% confidence interval [CI]: −0.30, 0.20; *p* = 0.6956). Daily LSMean estimates of the FLU‐PRO total score during the first 7 days of the RSV‐ARI episode showed a trend (i.e., numerical difference) for participants to recover more rapidly from their symptoms in the RSVPreF3 OA group compared with the Placebo group (Figure S2).

**FIGURE 2:**
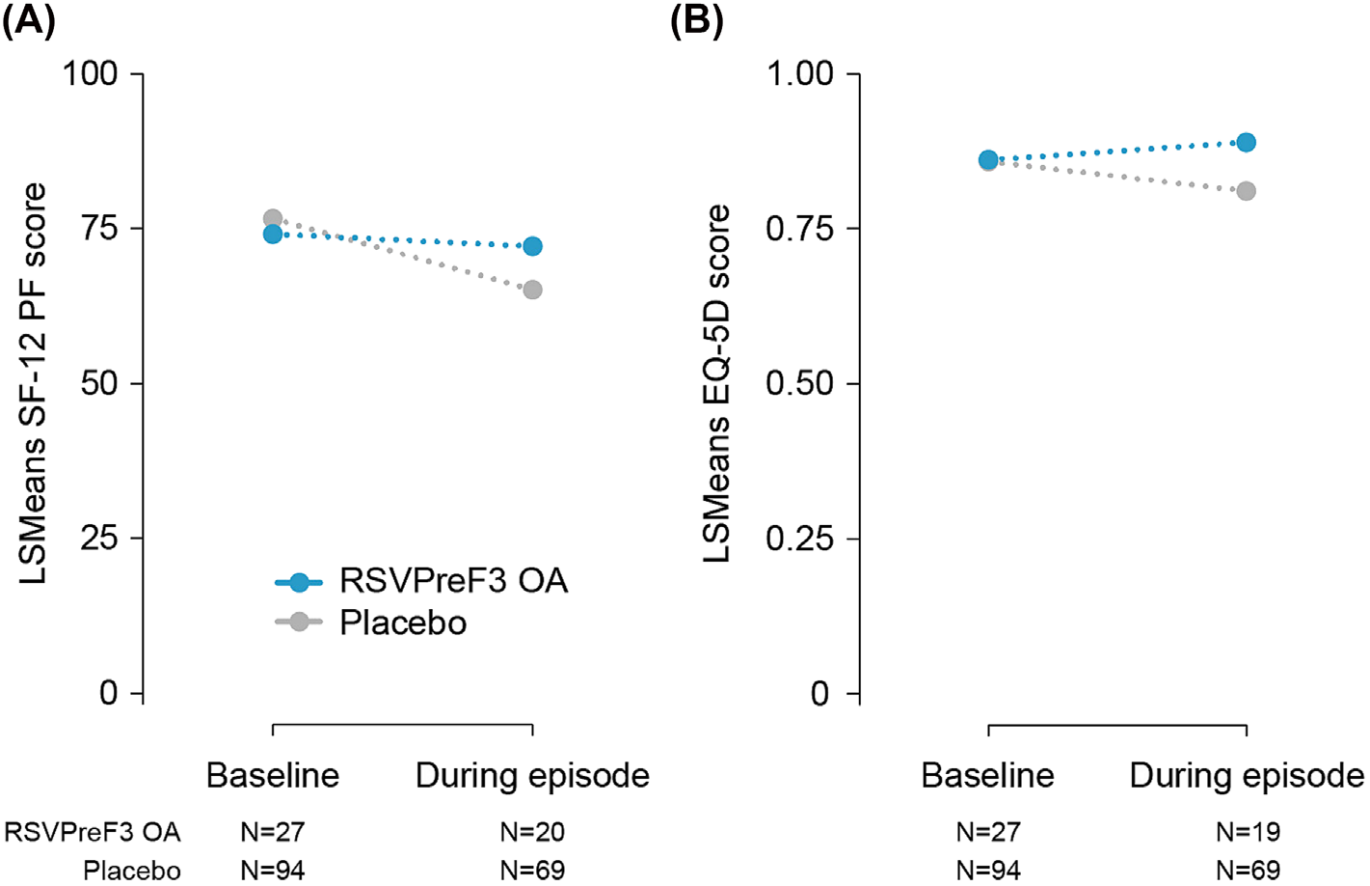
SF‐12 PF and EQ‐5D utility scores by assessment time and study group (mES RT‐PCR‐confirmed RSV‐ARI cohort). Least squares means were obtained from the longitudinal model featuring the baseline assessment and the assessment during the RSV‐ARI episode including terms for study group, timepoint, and timepoint by study group interaction term. A higher score indicates a higher level of functioning/quality of life. RSVPreF3 OA, participants receiving RSVPreF3 OA vaccine (27 participants with RSV‐ARI episodes); Placebo, participants receiving placebo (95 participants with RSV‐ARI episodes). EQ‐5D, EuroQol‐5 Dimension; LSMeans, Least squares means; mES, modified exposed set; *N*, number of observations; RSV‐ARI, respiratory syncytial virus–acute respiratory infection; RT‐PCR, reverse transcription polymerase chain reaction; SF‐12 PF, Short Form‐12 physical functioning.

The SF‐12 questionnaire was completed by 99.2% (121/122) of participants at baseline and by 73.0% (89/122) at the scheduled assessment during the RSV‐ARI episode. During the RSV‐ARI episode, the observed LSMeans (SE) of the SF‐12 PF scale were 72.16 (9.99) in the RSVPreF3 OA group and 65.16 (9.80) in the Placebo group (Table 2 and Figure 2). The difference in LSMeans between groups was 7.00 (95% CI: −9.86, 23.85; *p* = 0.4125). In the Placebo group, mean scores for the other seven domains of the SF‐12 questionnaire tended to decrease more during the RSV‐ARI episode versus baseline than in the RSVPreF3 OA group (Table S4).

**TABLE 2:** LSMeans for the FLU‐PRO total score, SF‐12 PF score, and the EQ‐5D utility score during the RSV‐ARI episode (mES RT‐PCR‐confirmed RSV‐ARI cohort).

Characteristic	RSVPreF3 OA *N* = 27	Placebo *N* = 95	LSMean difference (95% CI)	*p* value
FLU‐PRO total score				
*N* with data	24	76		
Baseline^a^	0.23	0.21		
During episode	0.76	0.81	−0.05 (−0.30, 0.20)	0.6956
SF‐12 physical functioning score				
*N* with data	20	69		
Baseline	74.15	76.58		
During episode	72.16	65.16	7.00 (−9.86, 23.85)	0.4125
EQ‐5D utility score				
*N* with data	19	69		
Baseline	0.8616	0.8586		
During episode	0.8896	0.8109	0.0786 (−0.0340, 0.1913)	0.1695

*Note*: A higher FLU‐PRO score indicates a higher level of symptom severity. A higher SF‐12 or EQ‐5D score indicates a higher level of functioning/quality of life.

Abbreviations: CI, confidence interval; EQ‐5D, EuroQol‐5 Dimension; FLU‐PRO, InFLUenza Patient‐Reported Outcome; LSMeans, least squares means, mES, modified exposed set; *N*, number of first RT‐PCR‐confirmed RSV‐ARI episodes; Placebo, participants receiving placebo; RSV‐ARI, respiratory syncytial virus–acute respiratory infection; RSVPreF3 OA, participants receiving RSVPreF3 OA vaccine; RT‐PCR, reverse transcription polymerase chain reaction; SF‐12 PF, Short Form‐12 physical functioning.

^a^ Mean score of the assessments at baseline.

The EQ‐5D questionnaire was completed by 99.2% (121/122) of participants at baseline and 72.1% (88/122) for the scheduled assessment during the RSV‐ARI episode. During the RSV‐ARI episode, the observed LSMeans (SE) for the EQ‐5D utility scores were 0.8896 (0.0719) in the RSVPreF3 OA group and 0.8109 (0.0736) in the Placebo group (Table 2 and Figure 2). The difference in LSMeans between groups was 0.0786 (95% CI: −0.0340, 0.1913; *p* = 0.1695).

The median (mean) duration of the RSV‐ARI episode was 11.0 days (12.8 days) in the RSVPreF3 OA group compared with 14.0 days (19.0 days) in the Placebo group. A medical visit (hospitalization or an otherwise unscheduled visit to/from medical personnel) during the RSV‐ARI episode was required for 29.6% (8/27) of the participants in the RSVPreF3 OA group compared with 40.0% (38/95) in the Placebo group. The VE against RSV‐confirmed ARI with medically attended visits was 79.0% (95% CI: 54.3 to 91.5). A medical visit during the RSV‐LRTD episode was required for 42.7% (3/7) of the participants in the RSVPreF3 OA group, compared with 60.0% (24/40) in the Placebo group, yielding a VE against RSV‐confirmed LRTD with medically attended visits of 87.5% (95% CI, 58.9 to 97.6).

## 4 DISCUSSION

In this manuscript, we present a statistically and clinically significant reduction in the median peak FLU‐PRO Chest/Respiratory scores during RSV‐ARI episodes in participants vaccinated with RSVPreF3 OA compared with placebo. Participants in the RSVPreF3 OA group had less severe chest/respiratory symptoms such as trouble breathing, chest tightness, and frequency and severity of cough, compared with participants in the Placebo group. The results suggest that the RSVPreF3 OA vaccine, in addition to preventing illness, attenuated the severity of RSV‐ARI‐associated symptoms in RSV breakthrough infections. This finding is in line with the efficacy results demonstrating higher VE against more severe RSV disease, that is, VE against severe RSV‐LRTD (94.1%) > VE against RSV‐LRTD (82.6%) > VE against RSV‐ARI (71.7%).^9^

Based on the peak score, no clear trends in reduced severity were observed for the FLU‐PRO domains except for the FLU‐PRO Chest/Respiratory domain (e.g., upper respiratory, gastrointestinal, and body/systemic symptoms) resulting in no overall difference for the FLU‐PRO total score. However, the trend for individuals to recover more rapidly from their symptoms in the RSVPreF3 OA group compared with the Placebo group is consistent with the trend of a reduced duration of the RSV‐ARI episode in the RSVPreF3 OA group.

The World Health Organization (WHO) defined healthy ageing as developing and maintaining functional ability to enable well‐being in older age.^19^, ^20^ In this study, during the RSV‐ARI episode, placebo recipients experienced a decrease of approximately 10 points from baseline in their SF‐12 PF score. Branche et al. reported that some older adults with RSV, in particular hospitalized individuals, demonstrate acute functional decline that may become prolonged.^8^ The difference in SF‐12 PF LSMean estimates between groups during the RSV‐ARI episode was seven points, suggesting that the impact of RSV on PF was lower in the vaccinated group. An MCID of 3.3 in the SF‐36 PF was estimated for participants with improving symptoms of lower extremities osteoarthritis,^21^ suggesting that the difference between the groups in the current study is clinically significant. A similar trend was observed in EQ‐5D utility values where the difference in LSMean estimates between groups was 0.0786. Considering that in patients with cancer, Pickard et al.^22^ showed MCID values ranging between 0.06 (the United States) and 0.09 (the United Kingdom), this finding also suggests that the decrease in health utility in the Placebo group compared with RSVPreF3 OA group is clinically relevant.

In addition, consistent trends were observed for the other SF‐12 domains suggesting that the impact of the RSV‐ARI episode on HRQoL domains was attenuated in the RSVPreF3 OA group. Reduction of severity of breakthrough disease after vaccination has also been observed for other vaccine‐preventable diseases such as influenza, pertussis, rotavirus, and herpes zoster.^23^, ^24^, ^25^, ^26^

To reduce the patient burden, and facilitate study setup for patients, we used the SF‐12 questionnaire rather than the SF‐36 questionnaire and limited the number of days for completion of daily questionnaires to a maximum of 14 days following the onset of the RSV episode. The SF‐36 could have provided better precision, and therefore less uncertainty, for domains such as the PF scale for which 10 items are included in the SF‐36 compared with two in the SF‐12. Furthermore, we included only one assessment with the SF‐12 and EQ‐5D questionnaires during the RSV‐ARI episode (i.e., at the ARI visit). As such, the estimates of the impact of the RSV‐ARI episode on HRQoL may be underestimated as the peak of the episode may have occurred before or after the ARI visit. Analyzing subgroups of individuals within a randomized clinical trial, that is, only those who develop disease, is subject to selection bias.^27^ Breakthrough disease is more likely to occur in individuals who at baseline have underlying conditions or those who are frail, for example, due to vaccine efficacy being lower.^28^ As such, the results presented here may underestimate the true effect of the vaccine in attenuating the severity of disease, as subjects with underlying conditions or those who are more frail may be more likely to have more severe disease. Finally, because of the high VE against RSV‐ARI, and a generally low incidence of RSV during the winter of 2022 as a result of preventive measures against COVID‐19, a lower‐than‐expected number of breakthrough cases were observed, limiting the statistical power to identify significant differences between the groups. As the study is still ongoing (i.e., to include RSV Seasons 2 and 3), at the end‐of‐study analysis, additional data will be available.

## 5 CONCLUSION

The impact of RSV‐ARI on health and well‐being is substantial in older adults. The Peak FLU‐PRO Chest/Respiratory scores suggest that the RSVPreF3 OA vaccine, in addition to preventing infection, attenuated the severity of RSV‐ARI‐associated symptoms in RSV breakthrough infections. The observed reduction in symptoms translated into numerical differences in reduced impact of RSV‐ARI on PF that did not reach statistical significance. Adult vaccination against RSV has the potential to reduce the burden of disease and to help maintain functioning, quality of life, and support healthy ageing in older adults.

## CONFLICT OF INTEREST STATEMENT

During this study, Eliazar Sabater Cabrera, Desmond Curran, Veronica Hulstrøm, Lusine Kostanyan, and Daniel Molnar were GSK employees. Eliazar Sabater Cabrera, Desmond Curran, Veronica Hulstrøm, Lusine Kostanyan, and Daniel Molnar hold shares in GSK. Isabel Maria Galan Melendez was an investigator for the study of RSV OA‐006 and declares to have received payments from GSK per contract, as well as equipment on loan and study materials. Silvia Narejos Pérez participated as principal investigator (PI) in clinical trials with different sponsors, including GSK and received financial support during the present manuscript. As a PI, she collaborated in the presentation of the results of clinical trials of vaccines and received financial support for attending meetings and/or travel. Laura Helman received payment for completing the study work and was given financial support for attending meetings and/or travel as an investigator. During the development of the study, John H. Powers III was a consultant for GSK and Vir on the clinical study design. Besides that, he received consulting fees from Adaptive Phage, Arrevus, Atheln, Bavarian Nordic, Cellularity, Eicos, Evofem, Eyecheck, Gilead, Mustang, OPKO, Otsuka, Resolve, Romark, Spine BioPharma, and UTIlity. He is an unpaid board member of Health Literacy Media. Mika Rämet declares that his institute was sponsored by GSK to perform the current study. Tino Schwarz has received honoraria for lecturing or advisory boards from Alexion, AstraZeneca, Bavarian Nordic, Biogen, Biontech, CSL Seqirus, GSK, Janssen‐Cilag, Merck‐Serono, Moderna, MSD, Novavax, Pfizer, Roche, Sanofi‐Aventis, and Takeda and for conducting clinical vaccine trials from GSK, Pfizer, Clover Biopharmaceuticals, and Serum Institute of India. Brigitte Stephan participated as investigator in clinical trials with different sponsors, including GSK. Sean Matthews, Lina Pérez Breva, Nathalie Roy, Dae Won Park, and Axel Schaefer have nothing to declare.

### PEER REVIEW

The peer review history for this article is available at https://www.webofscience.com/api/gateway/wos/peer-review/10.1111/irv.13236.

## TRADEMARK STATEMENT

AS01 is a trademark owned by or licensed to GSK.

## Supporting information


**Figure S1.** Timing of PRO assessments during the ARI episode. Participants were asked to complete the SF‐12 and EQ‐5D questionnaires at baseline, i.e., at the vaccination visit. The FLU‐PRO, PGI‐S and PGI‐C questionnaires were completed at visit 2 (i.e., 28–42 days after vaccination). Subsequently, after experiencing at least two ARI symptoms/signs for 24 hours, participants had to contact the site staff to plan an ARI site visit. Participants with suspected ARI were asked to complete the FLU‐PRO, the PGI‐S, and PGI‐C daily from the day of onset until the end of the ARI episode or for a maximum of 14 consecutive days. Participants completed the SF‐12 and EQ‐5D once during the ARI episode while attending the scheduled ARI visit.

**Figure S2.** Daily LSMeans estimates of the FLU‐PRO total score during the first 7 days from the onset of the first RT‐PCR confirmed RSV‐ARI episode (mES RT‐PCR confirmed RSV‐ARI cohort) by study group. A higher score indicates a greater severity of symptoms/problems.
FLU‐PRO, InFLUenza Patient‐Reported Outcome; LSMeans, Least Squares means; mES, modified exposed set; N: number of observations; RSV‐ARI, respiratory syncytial virus ‐ acute respiratory infection; RT‐PCR, reverse transcription polymerase chain reaction.

**Data S1.** Supporting Information.

**Table S1.** Change in FLU‐PRO total and Chest/Respiratory scores from previous day categorized by the magnitude of the corresponding change in Patient Global Impression of Severity (PGI‐S) (mES RT‐PCR‐ confirmed RSV‐ ARI cohort).

**Table S2.** Change in FLU‐PRO total and Chest/Respiratory scores from previous day categorized by the corresponding rating of the Patient Global Impression of Change (PGI‐C) (mES RT‐PCR‐ confirmed RSV‐ARI cohort).

**Table S3.** Summary statistics of the maximum (peak) FLU‐PRO domain scores during the first 7 days from the onset of the first RT‐PCR‐confirmed RSV‐ARI episode (mES RT‐PCR‐confirmed RSV‐ARI cohort).

**Table S4.** Mean (SD) SF‐12 domain scores at baseline and during the RT‐PCR‐confirmed RSV‐ARI episode (mES RT‐PCR‐confirmed RSV‐ARI cohort).

## Data Availability

Anonymized individual participant data and study documents can be requested for further research from www.clinicalstudydatarequest.com.

## References

[1] Villanueva DH , Arcega V , Rao M . Review of respiratory syncytial virus infection among older adults and transplant recipients. Ther Adv Infect Dis. 2022;9:20499361221091413. doi:10.1177/20499361221091413 35464624 PMC9019318

[2] Falsey AR , Hennessey PA , Formica MA , Cox C , Walsh EE . Respiratory syncytial virus infection in elderly and high‐risk adults. N Engl J Med. 2005;352(17):1749‐1759. doi:10.1056/NEJMoa043951 15858184

[3] McClure DL , Kieke BA , Sundaram ME , et al. Seasonal incidence of medically attended respiratory syncytial virus infection in a community cohort of adults ≥50 years old. PLoS ONE. 2014;9(7):e102586. doi:10.1371/journal.pone.0102586 25025344 PMC4099308

[4] Centers for Disease Control and Prevention . Transmission of RSV (respiratory syncytial virus). 2022. Accessed February 10, 2023. https://www.cdc.gov/rsv/about/transmission.html

[5] Centers for Disease Control and Prevention . RSV in older adults and adults with chronic medical conditions. 2022. Accessed February 10, 2023. https://www.cdc.gov/rsv/high-risk/older-adults.html

[6] Yu J , Powers JH 3rd , Vallo D , Falloon J . Evaluation of efficacy endpoints for a phase IIb study of a respiratory syncytial virus vaccine in older adults using patient‐reported outcomes with laboratory confirmation. Value Health. 2020;23(2):227‐235. doi:10.1016/j.jval.2019.09.2747 32113628

[7] Curran D , Cabrera ES , Bracke B , et al. Impact of respiratory syncytial virus disease on quality of life in adults aged ≥50 years: a qualitative patient experience cross‐sectional study. Influenza Other Respi Viruses. 2022;16(3):462‐473. doi:10.1111/irv.12929 PMC8983922 34981637

[8] Branche AR , Saiman L , Walsh EE , et al. Change in functional status associated with respiratory syncytial virus infection in hospitalized older adults. Influenza Other Respi Viruses. 2022;16(6):1151‐1160. doi:10.1111/irv.13043 PMC9530534 36069297

[9] Papi A , Ison MG , Langley JM , et al. Respiratory syncytial virus prefusion F protein vaccine in older adults. N Engl J Med. 2023;388(7):595‐608. doi:10.1056/NEJMoa2209604 36791160

[10] Powers JH , Guerrero ML , Leidy NK , et al. Development of the Flu‐PRO: a patient‐reported outcome (PRO) instrument to evaluate symptoms of influenza. BMC Infect Dis. 2016;16(1):1. doi:10.1186/s12879-015-1330-0 26729246 PMC4700740

[11] Powers JH III , Bacci ED , Leidy NK , et al. Performance of the inFLUenza Patient‐Reported Outcome (FLU‐PRO) diary in patients with influenza‐like illness (ILI). PLoS ONE. 2018;13(3):e0194180. doi:10.1371/journal.pone.0194180 29566007 PMC5863969

[12] Gandek B , Ware JE , Aaronson NK , et al. Cross‐validation of item selection and scoring for the SF‐12 Health Survey in nine countries: results from the IQOLA Project. International Quality of Life Assessment. J Clin Epidemiol. 1998;51(11):1171‐1178. doi:10.1016/S0895-4356(98)00109-7 9817135

[13] Ware J Jr , Kosinski M , Keller SD . A 12‐Item Short‐Form Health Survey: construction of scales and preliminary tests of reliability and validity. Med Care. 1996;34(3):220‐233. doi:10.1097/00005650-199603000-00003 8628042

[14] Rabin R , de Charro F . EQ‐5D: a measure of health status from the EuroQol Group. Ann Med. 2001;33(5):337‐343. doi:10.3109/07853890109002087 11491192

[15] Hurst NP , Kind P , Ruta D , Hunter M , Stubbings A . Measuring health‐related quality of life in rheumatoid arthritis: validity, responsiveness and reliability of EuroQol (EQ‐5D). Br J Rheumatol. 1997;36(5):551‐559. doi:10.1093/rheumatology/36.5.551 9189057

[16] Food and Drug Administration . Guidance for industry—patient‐reported outcome measures: use in medical product development to support labeling claims. 2009. Accessed September 14, 2023. https://www.fda.gov/media/77832/download 10.1186/1477-7525-4-79 PMC1629006 17034633

[17] Delgado‐Herrera L , Lasch K , Zeiher B , et al. Evaluation and performance of a newly developed patient‐reported outcome instrument for diarrhea‐predominant irritable bowel syndrome in a clinical study population. Therap Adv Gastroenterol. 2017;10(9):673‐687. doi:10.1177/1756283X17726018 PMC5598814 28932269

[18] Chan ISF , Bohidar NR . Exact power and sample size for vaccine efficacy studies. Commun Stat Theory Methods. 1998;27(6):1305‐1322. doi:10.1080/03610929808832160

[19] World Health Organization . WHO's work on the UN Decade of Healthy Ageing (2021‐2030). 2023. Accessed February 10, 2023. https://www.who.int/initiatives/decade-of-healthy-ageing

[20] Rudnicka E , Napierała P , Podfigurna A , Męczekalski B , Smolarczyk R , Grymowicz M . The World Health Organization (WHO) approach to healthy ageing. Maturitas. 2020;139:6‐11. doi:10.1016/j.maturitas.2020.05.018 32747042 PMC7250103

[21] Angst F , Aeschlimann A , Stucki G . Smallest detectable and minimal clinically important differences of rehabilitation intervention with their implications for required sample sizes using WOMAC and SF‐36 quality of life measurement instruments in patients with osteoarthritis of the lower extremities. Arthritis Rheum. 2001;45(4):384‐391. doi:10.1002/1529‐0131(200108)45:4<384::AID‐ART352>3.0.CO;2‐0 11501727 10.1002/1529-0131(200108)45:4<384::AID-ART352>3.0.CO;2-0

[22] Pickard AS , Neary MP , Cella D . Estimation of minimally important differences in EQ‐5D utility and VAS scores in cancer. Health Qual Life Outcomes. 2007;5(1):70. doi:10.1186/1477-7525-5-70 18154669 PMC2248572

[23] Curran D , Oostvogels L , Heineman T , et al. Quality of life impact of an adjuvanted recombinant zoster vaccine in adults aged 50 years and older. J Gerontol A Biol Sci Med Sci. 2019;74(8):1231‐1238. doi:10.1093/gerona/gly150 29955836 PMC6625590

[24] Ehrlich HJ , Singer J , Berezuk G , et al. A cell culture–derived influenza vaccine provides consistent protection against infection and reduces the duration and severity of disease in infected individuals. Clin Infect Dis. 2012;54(7):946‐954. doi:10.1093/cid/cir959 22267715 PMC3297649

[25] Kendrick P , Eldering G . A study in active immunization against pertussis. Am J Epidemiol. 1939;29(3):133‐153. doi:10.1093/oxfordjournals.aje.a118485

[26] Vesikari T , Giaquinto C , Huppertz H‐I . Clinical trials of rotavirus vaccines in Europe. Pediatr Infect Dis J. 2006;25(1):S42‐S47. doi:10.1097/01.inf.0000197565.45345.4e 16397428

[27] Callegaro A , Curran D , Matthews S . Burden‐of‐illness vaccine efficacy. Pharm Stat. 2020;19(5):636‐645. doi:10.1002/pst.2020 32220002 PMC9291914

[28] Andrew MK , Bowles SK , Pawelec G , et al. Influenza vaccination in older adults: recent innovations and practical applications. Drugs Aging. 2019;36(1):29‐37. doi:10.1007/s40266-018-0597-4 30411283

