# Supplementary figures and images for: The respiratory syncytial virus prefusion F protein vaccine attenuates the severity of respiratory syncytial virus‐associated disease in breakthrough infections in adults ≥60 years of age
Source: Influenza Other Respir Viruses. 2024 Feb 3;18(2):e13236. doi: 10.1111/irv.13236 (PMC10837780; doi:10.1111/irv.13236)

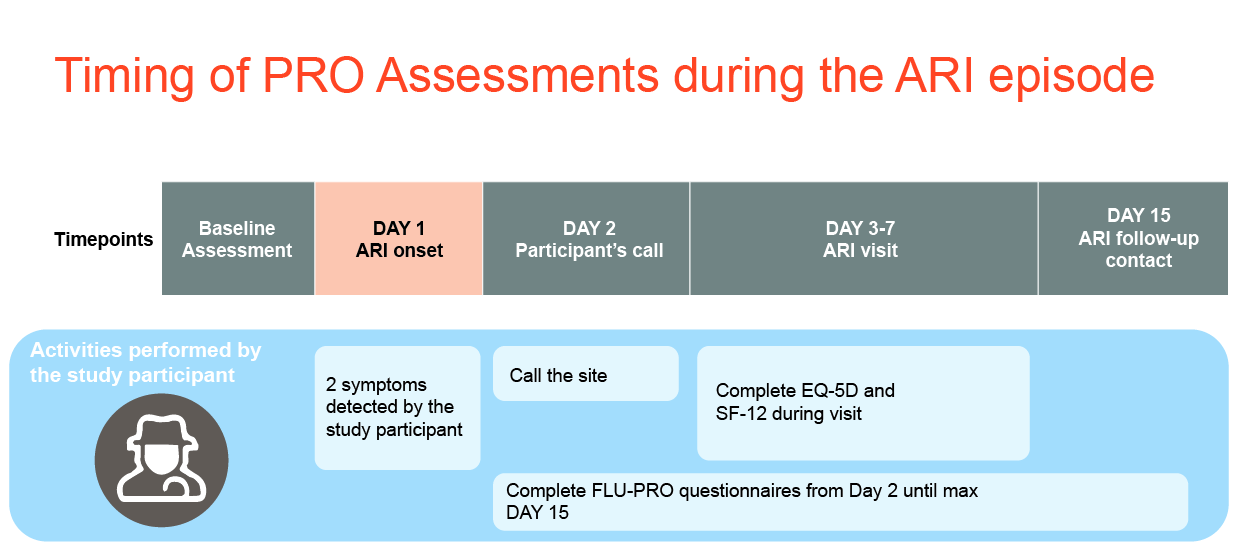

Supplement: Supplementary file 1 — Figure S1. Timing of PRO assessments during the ARI episode. Participants were asked to complete the SF‐12 and EQ‐5D questionnaires at baseline, i.e., at the vaccination visit. The FLU‐PRO, PGI‐S and PGI‐C questionnaires were completed at visit 2 (i.e., 28–42 days after vaccination). Subsequently, after experiencing at least two ARI symptoms/signs for 24 hours, participants had to contact the site staff to plan an ARI site visit. Participants with suspected ARI were asked to complete the FLU‐PRO, the PGI‐S, and PGI‐C daily from the day of onset until the end of the ARI episode or for a maximum of 14 consecutive days. Participants completed the SF‐12 and EQ‐5D once during the ARI episode while attending the scheduled ARI visit. [file IRV-18-e13236-s005.tif]

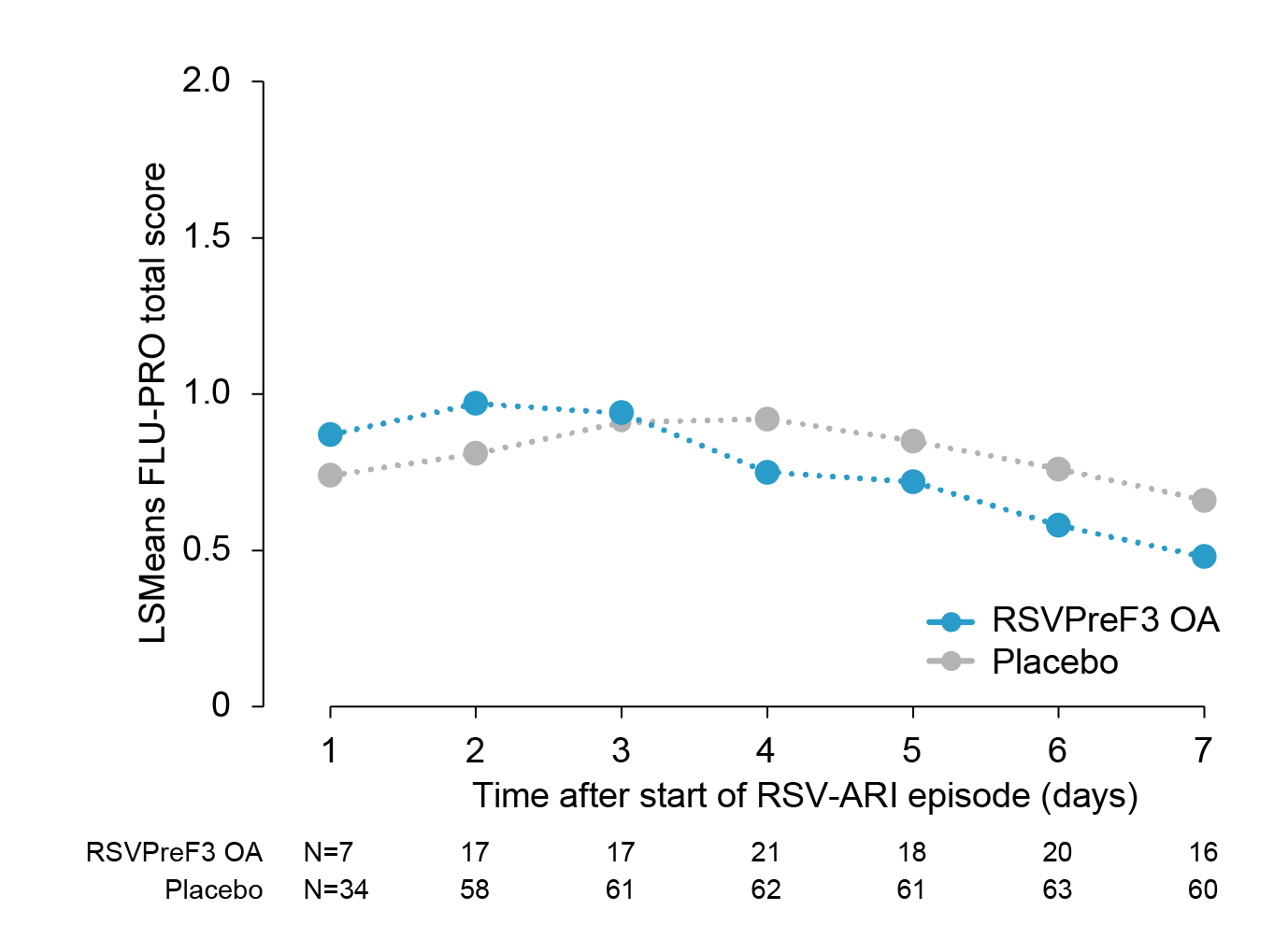

Supplement: Supplementary file 2 — Figure S2. Daily LSMeans estimates of the FLU‐PRO total score during the first 7 days from the onset of the first RT‐PCR confirmed RSV‐ARI episode (mES RT‐PCR confirmed RSV‐ARI cohort) by study group. A higher score indicates a greater severity of symptoms/problems. FLU‐PRO, InFLUenza Patient‐Reported Outcome; LSMeans, Least Squares means; mES, modified exposed set; N: number of observations; RSV‐ARI, respiratory syncytial virus ‐ acute respiratory infection; RT‐PCR, reverse transcription polymerase chain reaction. [file IRV-18-e13236-s007.tif]
