# Supplementary material for: The respiratory syncytial virus prefusion F protein vaccine attenuates the severity of respiratory syncytial virus‐associated disease in breakthrough infections in adults ≥60 years of age
Source: Influenza Other Respir Viruses. 2024 Feb 3;18(2):e13236. doi: 10.1111/irv.13236 (PMC10837780; doi:10.1111/irv.13236)
Supplement: Supplementary file 3 — Data S1. Supporting Information. [file IRV-18-e13236-s003.docx]

# SUPPLEMENTARY MATERIAL

Influenza patient reported outcome (FLU-PRO) response scales:

For vomiting or diarrhea, severity was assessed in terms of numerical frequency (0 times, 1 time, 2 times, 3 times, or 4 or more times); frequency of sneezing, coughing, and coughed up mucus or phlegm was evaluated on a scale from 0 (never) to 4 (always), while the remaining 27 items were evaluated on a scale from 0 (not at all), 1 (a little bit), 2 (somewhat), 3 (quite a bit), to 4 (very much).

**Members of the AReSVi-006 Study Group** (names are listed alphabetically): Adams, Mark; Adams, Michael; Akite, Elaine Jacqueline; Alt, Ingrid; Andrews, Charles; Antonelli-Incalzi, Rafaelle; Asatryan, Asmik; Athan, Eugene; Bahrami, Ghazaleh; Bargagli, Elena; Bhorat, Qasim; Bird, Paul; Borowy, Przemyslaw; Boutry, Celine; Brotons Cuixart, Carles; Browder, David; Brown, Judith; Buntinx, Erik; Cameron, Donald; Campora, Laura; Chinsky, Kenneth; Choi, Melissa; Choo, Eun-Ju; Collete, Delphine; Corral Carrillo, Maria; David, Marie-Pierre; Davis, Matthew G; de Heusch, Magali; de Looze, Ferdinandus; De Meulemeester, Marc; De Negri, Ferdinando; De Schrevel, Nathalie; DeAtkine, David; Dedkova, Viktoriya; Descamps, Dominique; Dezutter, Nancy; Dzongowski, Peter; Eckermann, Tamara; Essink, Brandon; Faulkner, Karen; Feldman, Robert; Ferguson, Murdo; Fissette, Laurence; Fuller, Gregory; Gentile, Ivan; Ghesquiere, Wayne; Grimard, Doria; Gruselle, Olivier; Halperin, Scott; Heer, Amardeep; Hotermans, Andre; Ison, Michael G; Jelinek, Tomas; Kamerbeek, Jackie; Kim, Hyo Youl; Kimmel, Murray; Koch, Mark; Kokko, Satu; Koski, Susanna; Kotb, Shady; Lalueza, Antonio; Langley, Joanne M; Lee, Dong-Gun; Lee, Jin-Soo; Leroux-Roels, Isabel; Lins, Muriel; Lombaard, Johannes; Mahomed, Akbar; Malerba, Mario; Marechal, Celine; Martinon-Torres, Federico; Martinot, Jean-Benoit; Masuet-Aumatell, Cristina; McNally, Damien; Medina Pech, Carlos Eduardo; Mendez Galvan, Jorge; Mesaros, Narcisa Elena; Mesotten, Dieter; Mitha, Essack; Mngadi, Kathryn; Moeckesch, Beate; Montgomery, Barnaby; Murray, Linda; Nally, Rhiannon; Newberg, Joseph; Nugent, Paul; Ochoa Mazarro, Dolores; Oda, Harunori; Olivier, Aurelie; Orso, Maurizio; Ortiz Molina, Jacinto; Pak, Tatiana; Papi, Alberto; Patel, Meenakshi; Patel, Minesh; Pedro Pijoan, Anna Maria; Perez Vera, Merce; Perez, Alberto Borobia; Pileggi, Claudia; Pregliasco, Fabrizio; Pretswell, Carol; Quinn, Dean; Reynolds, Michele; Romanenko, Viktor; Rosen, Jeffrey; Ruiz Antoran, Belen; Sakata, Hideaki; Sauter, Joachim; Sein Anand, Izabela; Serra Rexach, Jose Antonio; Shu, David; Siig, Andres; Simon, William; Smakotina, Svetlana; Steenackers, Katie; Tafuri, Silvio; Takazawa, Kenji; Tellier, Guy; Terryn, Wim; Tharenos, Leslie; Thomas, Nick; Toursarkissian, Nicole; Ukkonen, Benita; Vale, Noah; Van der Wielen, Marie; Van Landegem, Pieter-Jan; van Zyl‑Smit, Richard N; Vanden Abeele, Carline; Verheust, Celine; Vermeersch, Lode; Vicco, Miguel; Vitale, Francesco; Voloshyna, Olga; White, Judith; Wie, Seong-Heon; Wilson, Jonathan; Ylisastigui, Pedro.
