# Supplementary material for: The respiratory syncytial virus prefusion F protein vaccine attenuates the severity of respiratory syncytial virus‐associated disease in breakthrough infections in adults ≥60 years of age
Source: Influenza Other Respir Viruses. 2024 Feb 3;18(2):e13236. doi: 10.1111/irv.13236 (PMC10837780; doi:10.1111/irv.13236)
Supplement: Supplementary file 4 — Table S1. Change in FLU‐PRO total and Chest/Respiratory scores from previous day categorized by the magnitude of the corresponding change in Patient Global Impression of Severity (PGI‐S) (mES RT‐PCR‐ confirmed RSV‐ ARI cohort). [file IRV-18-e13236-s001.docx]

# Supplementary Table S1. Change in FLU-PRO total and Chest/Respiratory scores from previous day categorized by the magnitude of the corresponding change in Patient Global Impression of Severity (PGI-S) (mES RT-PCR- confirmed RSV- ARI cohort)

|  |  | **Change in PGI-S relative to previous day** | | | | | | | | | | |
| --- | --- | --- | --- | --- | --- | --- | --- | --- | --- | --- | --- | --- |
|  |  | **Worsening** | | | | **No change** | | | **Improvement** | | | |
| FLU-PRO | | **4** | **3** | **2** | **1** | |  | **1** | | **2** | **3** | **4** |
| Total score | | | | | | | | | | | | |
|  | N | 0 | 1 | 5 | 48 | | 268 | 60 | | 8 | 1 | 0 |
|  | Mean |  | 1.59 | 0.50 | 0.21 | | -0.04 | -0.26 | | -0.09 | -0.53 |  |
| Chest score | | | | | | | | | | | | |
|  | N | 0 | 1 | 5 | 48 | | 268 | 60 | | 8 | 1 | 0 |
|  | Mean |  | 2.57 | 0.54 | 0.45 | | -0.03 | -0.26 | | -0.01 | -0.29 |  |

Note 1: The change in FLU-PRO total and Chest/Respiratory scores is the change relative to the scores on the previous day. The change can only be calculated between questionnaires on consecutive days. If either score is missing, the change is missing.

Note 2: Improvement of one point in the severity of symptoms measured by the PGI-S is associated with a mean change in both the Flu-PRO total score and the FLU-PRO chest score of 0.26. (grey background)

Note 3: Participants in both study groups are included in this table.

Note 4: Only assessments between day 1 and day 7 inclusive are included in this analysis.

FLU-PRO, InFLUenza Patient-Reported Outcome; mES, modified exposed set; N, number of occurrences by category; RT-PCR, reverse transcription polymerase chain reaction.
