# Supplementary material for: The respiratory syncytial virus prefusion F protein vaccine attenuates the severity of respiratory syncytial virus‐associated disease in breakthrough infections in adults ≥60 years of age
Source: Influenza Other Respir Viruses. 2024 Feb 3;18(2):e13236. doi: 10.1111/irv.13236 (PMC10837780; doi:10.1111/irv.13236)
Supplement: Supplementary file 5 — Table S2. Change in FLU‐PRO total and Chest/Respiratory scores from previous day categorized by the corresponding rating of the Patient Global Impression of Change (PGI‐C) (mES RT‐PCR‐ confirmed RSV‐ARI cohort). [file IRV-18-e13236-s004.docx]

# Supplementary Table S2. Change in FLU-PRO total and Chest/Respiratory scores from previous day categorized by the corresponding rating of the Patient Global Impression of Change (PGI-C) (mES RT-PCR- confirmed RSV-ARI cohort).

|  | **Change in FLU-PRO relative to previous day** | | | |  |
| --- | --- | --- | --- | --- | --- |
| **PGI-C** | **Total score** | | **Chest/Respiratory score** | |  |
|  | **N** | **Mean** | **N** | **Mean** | |
| Much better | 38 | -0.14 | 38 | -0.18 | |
| Somewhat better | 43 | -0.17 | 43 | -0.17 | |
| A little better | 74 | -0.19 | 74 | -0.13 | |
| About the same | 156 | -0.03 | 156 | 0.01 | |
| A little worse | 54 | 0.17 | 54 | 0.24 | |
| Somewhat worse | 22 | 0.14 | 22 | 0.25 | |
| Much worse | 12 | 0.31 | 12 | 0.70 | |

Note 1: The change in FLU-PRO total and Chest/Respiratory scores is the change relative to the scores on the previous day. Improvement of one category in the severity of symptoms measured by the PGI-C (i.e., A little better) was associated with a mean change in the Flu-PRO total score and the FLU-PRO Chest/Respiratory score of 0.19 and 0.13 respectively (both were less than the value of 0.26 in Supplementary Table S1, grey background).

Note 2: Participants in both study groups are included in this table.

Note 3: Only assessments between day 1 and day 7 are included in this analysis.

FLU-PRO, InFLUenza Patient-Reported Outcome; mES, modified exposed set; N, number of occurrences by category; RT-PCR, reverse transcription polymerase chain reaction.
