# Supplementary material for: The respiratory syncytial virus prefusion F protein vaccine attenuates the severity of respiratory syncytial virus‐associated disease in breakthrough infections in adults ≥60 years of age
Source: Influenza Other Respir Viruses. 2024 Feb 3;18(2):e13236. doi: 10.1111/irv.13236 (PMC10837780; doi:10.1111/irv.13236)
Supplement: Supplementary file 6 — Table S3. Summary statistics of the maximum (peak) FLU‐PRO domain scores during the first 7 days from the onset of the first RT‐PCR‐confirmed RSV‐ARI episode (mES RT‐PCR‐confirmed RSV‐ARI cohort). [file IRV-18-e13236-s002.docx]

# Supplementary Table S3. Summary statistics of the maximum (peak) FLU-PRO domain scores during the first 7 days from the onset of the first RT-PCR-confirmed RSV-ARI episode (mES RT-PCR-confirmed RSV-ARI cohort).

|  | **RSVPreF3 OA**  **N=27** | | **Placebo**  **N=95** | |
| --- | --- | --- | --- | --- |
|  | **Median (IQR)** | **Mean (SD)** | **Median (IQR)** | **Mean (SD)** |
| **Nose** | 2.13 (1.50–2.88) | 2.17 (0.89) | 2.25 (1.75–2.58) | 2.21 (0.88) |
| **Throat** | 1.67 (0.50–2.50) | 1.62 (1.20) | 1.67 (0.67–2.00) | 1.48 (1.08) |
| **Eyes** | 0.83 (0.00–1.50) | 0.92 (0.88) | 0.67 (0.00–1.00) | 0.82 (0.91) |
| **Gastrointestinal** | 0.00 (0.00–0.38) | 0.25 (0.42) | 0.00 (0.00–0.50) | 0.26 (0.39) |
| **Body Systemic** | 0.86 (0.27–1.53) | 1.01 (0.83) | 1.00 (0.55–1.64) | 1.14 (0.80) |
| **Total score** | 1.00 (0.50–1.65) | 1.09 (0.70) | 1.10 (0.74–1.71) | 1.22 (0.62) |

The maximum score is the highest (peak) score observed during the first seven days of the first episode.

A higher score indicates a higher level of symptom severity.

RSVPreF3 OA, participants receiving RSVPreF3 OA vaccine; Placebo, participants receiving placebo.

FLU-PRO, InFLUenza Patient-Reported Outcome; IQR, inter-quartile range; mES, modified exposed set; N, number of first RT-PCR-confirmed RSV-ARI episodes; RSV-ARI, respiratory syncytial virus - acute respiratory infection; RT-PCR, reverse transcription polymerase chain reaction; SD, standard deviation.
