# Supplementary material for: The respiratory syncytial virus prefusion F protein vaccine attenuates the severity of respiratory syncytial virus‐associated disease in breakthrough infections in adults ≥60 years of age
Source: Influenza Other Respir Viruses. 2024 Feb 3;18(2):e13236. doi: 10.1111/irv.13236 (PMC10837780; doi:10.1111/irv.13236)
Supplement: Supplementary file 7 — Table S4. Mean (SD) SF‐12 domain scores at baseline and during the RT‐PCR‐confirmed RSV‐ARI episode (mES RT‐PCR‐confirmed RSV‐ARI cohort). [file IRV-18-e13236-s006.docx]

# Supplementary Table S4. Mean (SD) SF-12 domain scores at baseline and during the RT-PCR-confirmed RSV-ARI episode (mES RT-PCR-confirmed RSV-ARI cohort).

|  |  | | **RSVPreF3 OA**  **N=27** | **Placebo**  **N=95** |
| --- | --- | --- | --- | --- |
| **Physical Functioning** | | Baseline | 72.2 (32.8) | 74.5 (27.9) |
|  | | During episode | 71.3 (29.6) | 62.7 (32.8) |
| **Role Physical** | | Baseline | 79.2 (26.6) | 77.4 (25.0) |
|  | | During episode | 66.9 (32.3) | 54.9 (32.4) |
| **Bodily Pain** | | Baseline | 79.6 (26.9) | 77.1 (27.4) |
|  | | During episode | 80.0 (27.6) | 68.1 (32.1) |
| **General Health** | | Baseline | 70.0 (28.3) | 66.2 (24.3) |
|  | | During episode | 58.0 (30.6) | 50.3 (29.8) |
| **Vitality** | | Baseline | 63.9 (23.3) | 68.7 (20.9) |
|  | | During episode | 48.8 (25.0) | 44.6 (27.1) |
| **Social Functioning** | | Baseline | 92.6 (22.8) | 90.7 (18.7) |
|  | | During episode | 66.3 (35.6) | 62.7 (36.5) |
| **Role Emotional** | | Baseline | 92.6 (14.4) | 87.1 (20.1) |
|  | | During episode | 82.5 (27.9) | 76.1 (31.6) |
| **Mental Health** | | Baseline | 77.3 (17.7) | 81.8 (17.2) |
|  | | During episode | 76.3 (15.7) | 73.6 (20.5) |

RSVPreF3 OA, participants receiving RSVPreF3 OA vaccine; Placebo, participants receiving placebo.

mES, modified exposed set; N, number of first RT-PCR confirmed RSV-ARI episodes; RSV-ARI, respiratory syncytial virus - acute respiratory infection; RT-PCR, reverse transcription polymerase chain reaction; SD, standard deviation; SF-12, Short Form-12.
